# 

**Published:** 1878-05-20

**Authors:** 


					﻿VOL. VIII.	MAY 20, 1878.	No. 5.
£
w
H
Wh
rr\
H-i
1-3
<1
o
I—i
Q
r-r~l
H-i
s
Pd
ft
o
I*
g
§
I HK
o
QD
h—I
i. m
o-
£
t—<
o
M
r—i
HH i
H
H
£
W
co
W
IX
W
UQ
<5
H
X
THE SOUTHERN
MEDICAL RECORD.
Ji JAonthly Journal of Practical J^edic.in-Et-^
/,«----- '	’ (mDEX&y
Terms : 8*2 OO per annum in advance. Club Kates, 3 copies
$8.00. Single copies 80 cents.
“QUICQUID PRJECIPIES ESTO BREVIS.”
GO
PC
o
ft
Q
O
a
H
a
>
HH
o
iz;
GO
►—<
ft
S3
a
H
H-1
Q
I—I
H
ft
te
<G0
GO
Q
ft
t—i
Q
i—i
H
ft
ft
ESTABLISHED IN 1870.
ATLANTA, GA.: •
Sunny South Steam Publishing House.
Address R. C. WORD, M. D., Managing Editor, Atlanta, Ga.
				

